# 

**Published:** 1969-04

**Authors:** 


April 1969

### The Bristol Medico-Chirurgical Journal

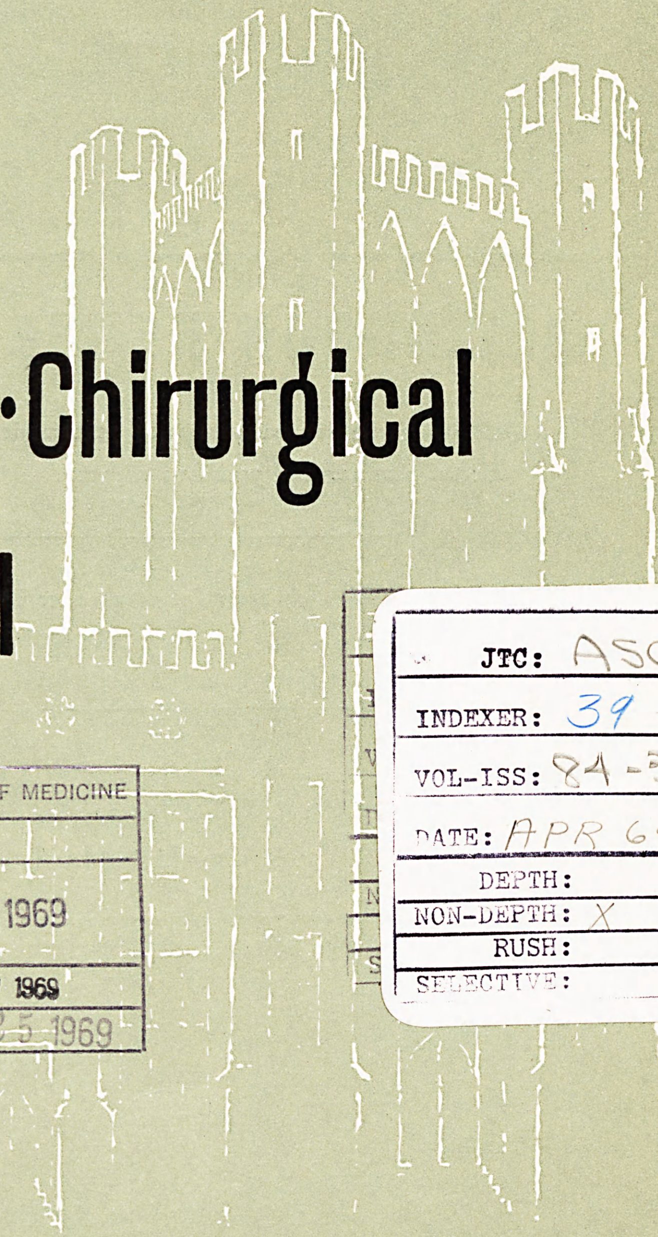

NATIONAL LIBRARY OF MEDICINE

RECD. MAY 15 1969

INDEX MAY 29 1969

INPUT JUN 25 1969

JTC: ASG
INDEXER: 39
VOL-ISS: 84-310
DATE: APR 69
DEPTH:
NON-DEPTH: X
RUSH:
SELECTIVE:

INCORPORATING THE MEDICAL JOURNAL OF THE SOUTH WEST

INDEX MEDICUS

Vol. 84 (*ii*) No 310

